# Prevalence and correlates of sexual intimate partner violence among trans women in the San Francisco Bay Area

**DOI:** 10.3389/fgwh.2025.1524148

**Published:** 2025-07-04

**Authors:** Glenn-Milo Santos, Willi McFarland, Erin C. Wilson

**Affiliations:** ^1^ Department of Community Health Systems, University of California, San Francisco, CA, United States; ^2^ San Francisco Department of Public Health, Center for Public Health Research, San Francisco, CA, United States; ^3^ Department of Epidemiology and Biostatistics, University of California, San Francisco, CA, United States

**Keywords:** transgender, intimate partner violence, mental health, substance use, violence

## Abstract

**Background:**

Intimate partner violence (IPV), including sexual IPV, is a significant public health issue with serious mental, physical, and economic consequences. Trans women are disproportionately affected by sexual IPV. However, research on factors associated with sexual IPV is limited among trans women. This study seeks to identify factors associated with sexual IPV in a large cohort of trans women in the San Francisco Bay Area.

**Methods:**

We conducted a secondary data analysis of data from the Trans*National cohort study (2016–2017), which enrolled 629 trans women via respondent-driven sampling; we conducted bivariate and multivariable logistic regression analyses to examine correlates of lifetime history of sexual IPV.

**Results:**

The prevalence of lifetime sexual IPV was 36%, and bivariate analyses identified several factors associated with sexual IPV, including inconsistent hormone use, non-prescribed hormone use, sex work, polysubstance use, depression, posttraumatic stress disorder, suicidality, homelessness, and discrimination. Multivariable models revealed significant associations between sexual IPV and psychosocial factors, such as substance use, mental health diagnoses, and experiences of violence and discrimination.

**Conclusion:**

These findings are consistent with the substance abuse, violence, and HIV/AIDS syndemic framework, underscoring the interconnectedness of these conditions among trans women. In addition, the findings suggest that disruptions in access to gender-affirming care may be a negative consequence of sexual IPV. These results also highlight the urgent need for integrated approaches to address the mental health, substance use, and HIV prevention needs of trans women who experience sexual IPV. Interventions that address structural discrimination and provide holistic support are necessary to improve the health and wellbeing of trans women survivors of sexual IPV.

## Introduction

Intimate partner violence (IPV)—defined as any form of abuse or aggression that occurs within a romantic or intimate relationship, including physical, psychological, and sexual violence—is a significant public health issue with serious mental (1, 2), physical (3, 4), and economic (5) impacts on victims. Moreover, sexual IPV—a type of IPV involving non-consensual sexual contact from a person's intimate partner, can also increase vulnerability to sexually transmitted infections (STI), including HIV (6), while also functioning as a barrier to engagement in HIV care and prevention (7).

Although the IPV research among trans women is relatively limited compared with studies in cisgender populations, available data demonstrate that IPV and sexual IPV disproportionately impact trans women compared with cisgender individuals (8). The 2015 US Transgender Survey of nearly 28,000 trans individuals found that 54% of respondents experienced IPV, and 19% of respondents experienced sexual IPV (9). Sexual IPV among trans women is often exacerbated by intersecting factors such as transphobia and systemic discrimination (8, 10, 11). For example, trans women may be uniquely vulnerable to sexual IPV because their gender identity and expression can increase their social and economic isolation, making them potentially more dependent on abusive partners for survival (8, 12). The transphobia, stigma, and discrimination faced by trans women can also impede their ability to seek help and support from medical and service providers for domestic violence and law enforcement (8, 13). Furthermore, trans women of color may be less likely to engage with law enforcement due to intersecting stigmas (i.e., racism, xenophobia, and deportation threats) (14).

A systematic review identified 74 unique studies that have examined IPV among trans participants, including sexual IPV (8). This study observed that the median lifetime prevalence of sexual IPV among transgender individuals was 25% across 14 studies, and the prevalence of sexual IPV in the past year was 10.8% across seven studies (8). Relative to cisgender individuals, transgender individuals are 2.5 times more likely to experience sexual IPV based on pooled data from 15 studies reviewed (8). However, none of these studies reported the demographic and health correlates associated with sexual IPV, underscoring a significant gap in public health research (8). Prior studies have documented that lifetime IPV experience was correlated with socio-ecological factors (e.g., experiencing homelessness, stigma, and discrimination) and other health conditions (e.g., mental health issues, suicidality, and substance use). These studies are consistent with syndemic theory, which posits that multiple psychosocial conditions often cluster and synergistically interact, but it is unclear whether these factors are correlated specifically with sexual IPV (8). Moreover, only some of the reviewed literature focused specifically on trans women, underscoring limitations in existing IPV studies (8). In addition, although access to gender-affirming care has been linked to safety for trans women as they are not as easily identifiable in public as trans, potentially reducing their risk for discrimination, harassment, and violence (15, 16), it is unclear whether access to gender-affirming care is linked to sexual IPV.

Understanding trans women's specific vulnerabilities and health conditions linked to sexual IPV can promote a public health response and inform the development of interventions for trans women harmed by sexual IPV. Therefore, we seek to close this gap in the literature by examining the factors associated with sexual IPV in a large, diverse sample of trans women in the San Francisco Bay Area. We hypothesize that we will observe consistent correlations between the factors linked to any IPV (8) observed in the systematic review of literature of studies with trans individuals including trans women, with our outcome of sexual IPV in the present study, and that the socio-ecological model and syndemic framework can help describe conditions related specifically to sexual IPV among trans women. In exploratory analyses, we also examine the associations between sexual IPV and gender-affirming care among trans women. Specifically, in line with syndemic theory, we posit that the same factors that drive disparities among trans women (e.g., stigma, discrimination, and socioeconomic disparities) may lead to the co-occurrence of negative psychosocial conditions, including the co-occurrence of lack of gender-affirming care and experiencing sexual IPV. Further, we acknowledge that bidirectional relationships between these syndemic conditions may exist, and that experiences of sexual IPV may also lead to the development of these factors.

## Methods

### Study design

This is a secondary data analysis of the baseline survey of the Trans National Study of trans women in the San Francisco Bay Area, conducted between 2016 and 2017. The procedures for this study have been previously described (17). In brief, a sample of 629 trans women was enrolled in a cohort study, Trans*National, to examine HIV incidence (17). The Trans*National Study used respondent-driven sampling (RDS), a peer-network-based recruitment, and a chain-referral approach for populations that are harder to reach using traditional recruitment strategies (17). Eligibility criteria for the study included being aged 18 years or older, assigned male sex at birth and not currently identifying as male in gender, and living in the San Francisco Bay Area (17). All participants provided informed consent. The University of California, San Francisco, Institutional Review Board approved all study procedures.

### Study measures

The study used a standardized questionnaire administered with computer-assisted program interviews (CAPI), and a more exhaustive list of measures has been previously reported (17). Measures included demographic characteristics (e.g., age, gender identity, race/ethnicity, current living situation, education, sexual orientation, and marital status), behaviors in the past 6 months (e.g., number of sexual partners, frequency of sexual intercourse, condomless sex, substance use, sex work), gender-affirming services (hormone use, surgery), and health conditions (e.g., mental health, suicidality, STI). The study outcome for the present study is sexual IPV, which was measured using the following question: “Has a partner ever hurt you sexually or made you do something sexual that you did not want to do? (Yes/No).”

### Statistical analyses

Given the large number of measures in the study, we used a confirmatory analytic approach. We focused our analysis on factors previously identified as correlates of IPV in general in a systematic review of the literature among trans individuals, inclusive of trans women (8). These included factors that may be considered as potential antecedents that may put trans women at increased risk for sexual IPV through a socio-ecological model lens: homelessness (9), immigration status (9), incarceration (18), and lower educational attainment (19). We also examined the correlation between sexual IPV and psychosocial conditions consistent with the substance abuse, violence, and HIV/AIDS (SAVA) syndemic framework, including substance use [recent substance use (20), polysubstance abuse (21), and substance use treatment (20)], mental health [depression (22), posttraumatic stress disorder (PTSD) (23), and avoidant coping (22)], violence [general victimization (21), gender-related victimization (18), and everyday discrimination (22)], and HIV sexual behaviors [greater number of sexual partners (23), transactional sex (9, 18, 24), STI diagnosis (23), and condomless sex (21, 23)]. In addition, as part of exploratory analyses, and in line with syndemic theory, which posits the clustering of negative psychosocial conditions, we are interested in the potential co-occurrence of sexual IPV and lack of access to gender-affirming services; we explored associations with factors such as inconsistent hormone use and access to surgery in this sample.

Chi-square, Wilcoxon rank-sum, and T-tests were used to compare differences between trans women who experienced sexual IPV and those who did not. Factors significantly associated with the outcome in bivariate analyses using an alpha cut-off of 0.10 were further examined in multivariable analyses. This process is similar to the approach previously proposed by others, which used a higher threshold for model building (25). To avoid the Table 2 fallacy, which is defined as misinterpreting results with covariates in multivariable models that are not central to the main hypotheses, we fitted models separately for the significant bivariate correlates above to estimate their association with our outcome (26, 27). This approach also mitigated potential concerns around multicollinearity, given the potential overlaps between these correlates. We adjusted each separate model for a limited number of covariates hypothesized to be potential confounders, including age, sexual orientation, HIV status, race and ethnicity, and education, consistent with prior studies (28, 29). We hypothesized that these covariates are associated with our exposures and outcome of interest, and we wanted to examine the magnitude of the relationship between our exposures and outcomes independent of these covariates (i.e., holding them constant in the model). All analyses were conducted using STATA version 18.0 (College Station, TX, USA).

## Results

### Study characteristics

The characteristics of the Trans*National Cohort of 629 trans women are summarized in Table 1. The study included a diverse sample of trans women (17% African American, 33% Latine, 5% Asian and Pacific Islander, and 16% reported another race or multiple races). The mean age of study participants was 40.5 (SD = 13). The overall prevalence of any IPV was 64% (400 out of 629), while the prevalence of sexual IPV in the overall sample was 36% (229 out of 629). Among the 400 participants who experienced any IPV, the majority (57%) had experienced sexual IPV (229 out of 400).

**Table 1:** Sociodemographic characteristics of trans women in San Francisco cross-sectional study, *N* = 629, 2016–2017.

Characteristic	*N*	%
Age, mean (standard deviation)	40.5 (13)	
Gender identity		
Female, woman, or transgender	572	90.9
Genderqueer/Genderfluid	23	3.7
Androgynous/Ambigender	3	0.5
Questioning	2	0.3
Other	29	4.6
Currently living full-time as gender identity		
No	25	4.0
Yes	602	95.7
Race/ethnicity		
White	182	28.9
Asian and Pacific Islander	29	4.6
Black/African American	107	17.0
Hispanic/Latinx	205	32.6
Other or Multiple	106	16.9
Current living situation		
Own or rent	295	46.9
Homeless/shelter	106	16.9
SRO	122	19.4
Residential treatment facility	17	2.7
Transitional/supportive housing	20	3.2
Couch surfing or other	69	11.0
Education		
Grades 1–8	33	5.2
Grades 9–11	91	14.5
Completed high school or General Educational Development (GED)	179	28.5
Some college, associate degree, or technical degree	208	33.1
Bachelor's degree	83	13.2
Any postgrad studies	35	5.6
Annual income		
<$12,060	362	57.6
$12,060–20,000	86	13.7
$20,000–30,000	84	13.4
$30,000–45,000	40	6.4
$45,000–70,000	22	3.5
$70,000–100,000	12	1.9
$100,000–150,000	12	1.9
>$150,000	11	1.7
At or below extremely low income limit for SF	488	77.6
Sexual orientation		
Straight/heterosexual	269	42.8
Gay/lesbian	89	14.1
Bisexual	96	15.3
Pansexual	52	8.3
Queer	73	11.6
Questioning	11	1.7
Other	36	5.7
Marital status		
Never married	436	69.3
Separated or divorced	89	14.1
Widowed	23	3.7
Married	47	7.5
Living together as married	34	5.4
Ever sex work	406	64.5

### Factors associated with sexual IPV

Tables 2 and 3 summarizes the results of the bivariate analyses comparing those with lifetime sexual IPV and those without. Factors associated with sexual IPV included the following: condomless sex (*p* = 0.34), intermittent hormone use (*p* = 0.006), non-prescribed hormone use (*p* < 0.001), pre-exposure prophylaxis (PrEP) use (*p* = 0.008), a greater number of sexual partners (*p* = 0.039), engaging in sex work (*p* < 0.001), a greater number of substances used (*p* = 0.002), participation in an alcohol or substance use treatment program (*p* = 0.004), receiving a depression diagnosis (*p* = 0.001), receiving a PTSD diagnosis (*p* < 0.001), receiving an anxiety diagnosis (*p* = 0.006), reporting prior suicide attempts (*p* < 0.001), prior suicidal thoughts (*p* < 0.001), binge drinking (*p* = 0.004), ever experiencing homelessness (*p* < 0.001), history of being undocumented (*p* = 0.008), experiencing a hate crime (*p* = 0.001), having lower income (*p* = 0.032), history of physical abuse (*p* < 0.001), and history of verbal abuse (*p* < 0.001). Other factors that met the alpha threshold to be considered for multivariable analyses include injection drug use and any substance use.

**Table 2:** Bivariable analyses of sociodemographic characteristics and sexual intimate partner violence among trans women in the San Francisco Bay Area, 2016–2017.

Characteristic		Sexual IPV *N* (%)		*p*-value
		No	Yes	
		*N* = 400	*N* = 229	
Participant age, median (IQR)		41.5 (30–52)	37 (28–50)	0.051
Sexual orientation	Straight/heterosexual	181 (45.4%)	88 (38.8%)	0.063
	Gay/lesbian	65 (16.3%)	24 (10.6%)	
	Bisexual	56 (14.0%)	40 (17.6%)	
	Pansexual	31 (7.8%)	21 (9.3%)	
	Queer	41 (10.3%)	32 (14.1%)	
	Other	25 (6.3%)	22 (9.7%)	
Race/ethnicity	White, Non-Hispanic/Latino	119 (29.8%)	63 (27.5%)	0.094
	Asian, Non-Hispanic/Latino	24 (6.0%)	5 (2.2%)	
	African American, Non-Hispanic/Latino	71 (17.8%)	36 (15.7%)	
	Other, Non-Hispanic/Latino	67 (16.8%)	39 (17.0%)	
	Hispanic/Latina	119 (29.8%)	86 (37.6%)	
Education level	College degree and beyond	80 (20.0%)	38 (16.6%)	0.13
	Less than HS	81 (20.2%)	43 (18.8%)	
	HS diploma/GED	120 (30.0%)	59 (25.8%)	
	Some college/technical degree	119 (29.8%)	89 (38.9%)	
Ever victim of a hate crime	No	235 (59.0%)	105 (45.9%)	0.001
	Yes	163 (41.0%)	124 (54.1%)	
Average gross annual income	Above low income level	25 (6.4%)	3 (1.3%)	0.032
	Low income	19 (4.8%)	10 (4.4%)	
	Very low income	34 (8.7%)	22 (9.6%)	
	Extremely low income	315 (80.2%)	194 (84.7%)	
Marital status	Never married	275 (68.8%)	161 (70.3%)	0.86
	Separated	21 (5.2%)	12 (5.2%)	
	Divorced	40 (10.0%)	16 (7.0%)	
	Widowed	14 (3.5%)	9 (3.9%)	
	Married	30 (7.5%)	17 (7.4%)	
	Living together as married	20 (5.0%)	14 (6.1%)	
Ever homeless	No	116 (29.0%)	35 (15.3%)	<0.001
	Yes	284 (71.0%)	194 (84.7%)	
Ever undocumented immigrant	No	38 (47%)	11 (23%)	0.008
	Yes	43 (53%)	36 (77%)	
History of incarceration	No	154 (38.6%)	76 (33.3%)	0.19
	Yes	245 (61.4%)	152 (66.7%)	
Sex work: ever	No	164 (41.1%)	57 (25.0%)	<0.001
	Yes	235 (58.9%)	171 (75.0%)	

**Table 3:** Bivariable analyses of clinical, HIV-related factors, and sexual intimate partner violence among trans women in the San Francisco Bay Area, 2016–2017.

Characteristic		Sexual IPV *N* (%)		*p*-value
		No	Yes	
		*N* = 400	*N* = 229	
Currently on hormones	No	66 (18.3%)	45 (21.6%)	0.33
	Yes	295 (81.7%)	163 (78.4%)	
Ever taken hormones	No	39 (9.8%)	21 (9.2%)	0.81
	Yes	361 (90.2%)	208 (90.8%)	
Intermittent hormone use	No	136 (37.6%)	55 (26.3%)	0.006
	Yes	226 (62.4%)	154 (73.7%)	
Ever non-prescribed hormones	No	198 (54.8%)	81 (38.8%)	<0.001
	Yes	163 (45.2%)	128 (61.2%)	
Any type of gender-affirming surgery	No surgery	189 (47.2%)	116 (50.7%)	0.41
	Any surgery	211 (52.8%)	113 (49.3%)	
Lab-rested HIV status	Negative	277 (69.6%)	166 (72.5%)	0.44
	Positive	121 (30.4%)	63 (27.5%)	
Most recent viral load count	Undetectable	72 (78%)	47 (89%)	0.12
	Detectable	20 (22%)	6 (11%)	
Taken PrEP before sex to prevent HIV	No	203 (89.8%)	111 (79.9%)	0.008
	Yes	23 (10.2%)	28 (20.1%)	
How many sex partners last 6 months, median (IQR)		1 (0–4)	2 (1–5)	0.039
Condomless sex	No	251 (62.7%)	124 (54.1%)	0.034
	Yes	149 (37.2%)	105 (45.9%)	
Sexually transmitted infections, past 6 months	No	260 (65.0%)	146 (63.8%)	0.75
	Yes	140 (35.0%)	83 (36.2%)	
Injection drug use, past 12 months	No	368 (92.2%)	202 (88.2%)	0.094
	Yes	31 (7.8%)	27 (11.8%)	
Binge drinking, past 12 months	No	255 (64.1%)	120 (52.4%)	0.004
	Yes	143 (35.9%)	109 (47.6%)	
Alcohol use disorder identification test (AUDIT)	Low risk	323 (81.4%)	176 (76.9%)	0.18
	High risk	74 (18.6%)	53 (23.1%)	
Any substance use last 12 months	No	196 (49.0%)	95 (41.5%)	0.069
	Yes	204 (51.0%)	134 (58.5%)	
Number of substances used, past 12 months, median (IQR)		0 (0–1)	0 (0–2)	0.002
Ever participated in alcohol/drug treatment program	No	250 (63.0%)	117 (51.1%)	0.004
	Yes	147 (37.0%)	112 (48.9%)	
Depression diagnosis	No	183 (45.9%)	75 (32.9%)	0.001
	Yes	216 (54.1%)	153 (67.1%)	
PTSD diagnosis	No	290 (72.7%)	127 (55.7%)	<0.001
	Yes	109 (27.3%)	101 (44.3%)	
Anxiety diagnosis	No	203 (50.9%)	90 (39.5%)	0.006
	Yes	196 (49.1%)	138 (60.5%)	
Attempted suicide	No	245 (61.4%)	93 (40.8%)	<0.001
	Yes	154 (38.6%)	135 (59.2%)	
Thoughts of suicide	No	142 (35.9%)	38 (16.6%)	<0.001
	Yes	254 (64.1%)	191 (83.4%)	
Verbally abused or harassed	No	68 (17.0%)	13 (5.7%)	<0.001
	Yes	332 (83.0%)	216 (94.3%)	
Physically abused or harassed	No	192 (48.2%)	49 (21.4%)	<0.001
	Yes	206 (51.8%)	180 (78.6%)	

**Figure 1:**
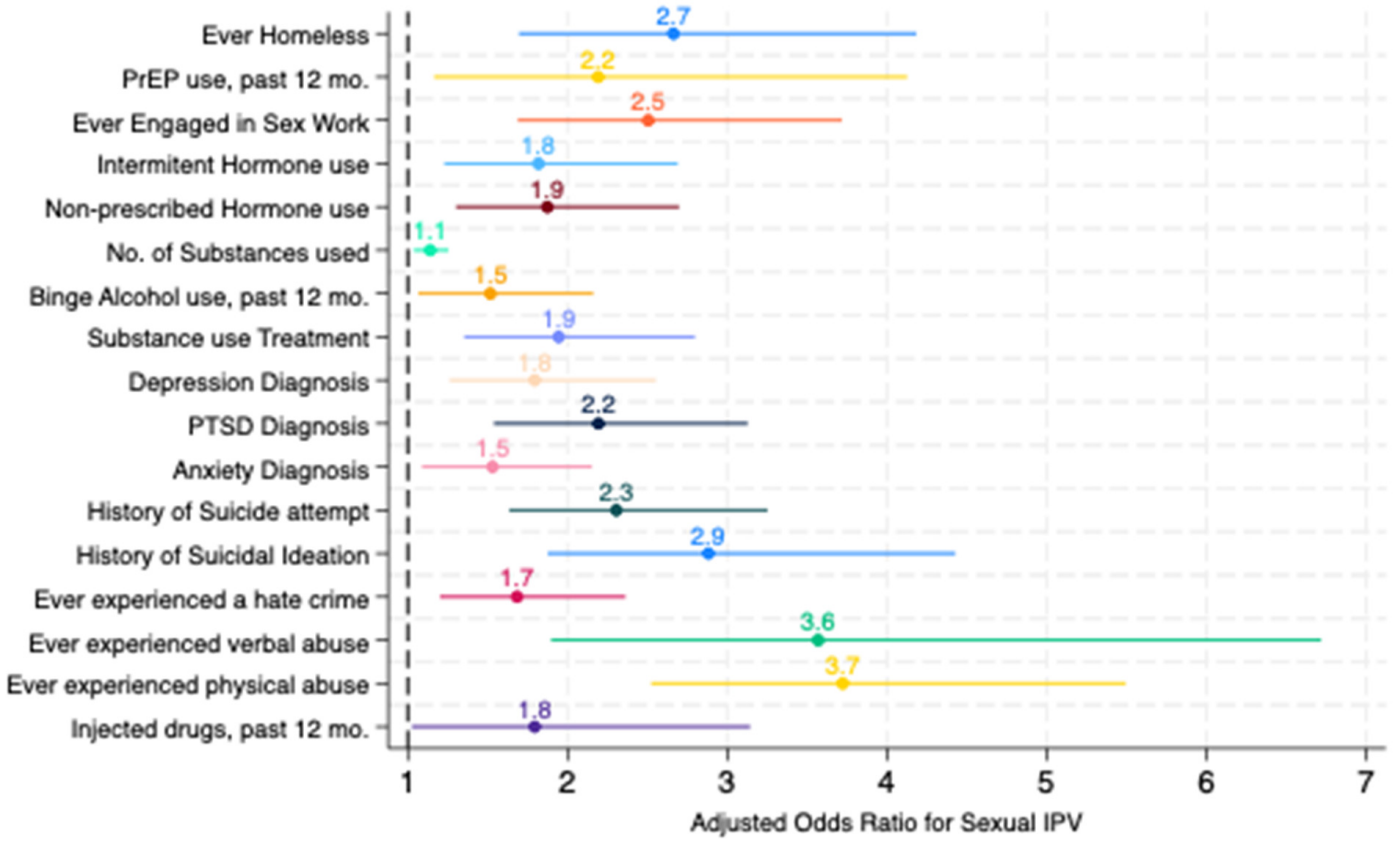
Results of multivariable logistic regression models for sexual intimate partner violence among trans women in the San Francisco Bay Area, 2016–2017. Note: each characteristic above was fit in separate regression models, adjusting for age, HIV status, race/ethnicity, and sexual orientation.

In multivariable logistic regression models (see Figure 1), factors associated with increased odds of sexual IPV victimization included reporting intermittent hormone use [adjusted odds ratio (aOR) = 1.82; 95% CI: 1.23–2.69], ever using non-prescribed hormones (aOR = 1.87; 95% CI: 1.30–2.70), taking PrEP (aOR = 2.19; 95% CI: 1.16–4.13), greater number of substances used in the past 12 months (aOR = 1.14; 95% CI: 1.04–1.25), ever participating in alcohol or substance use treatment programs (aOR = 1.94; 95% CI:1.35–2.80), receiving a depression diagnosis (aOR = 1.79; 95% CI: 1.26–2.55), receiving a PTSD diagnosis (aOR = 2.19; 95% CI: 1.54–3.13), receiving an anxiety diagnosis (aOR = 1.53; 95% CI: 1.09–2.15), reporting prior suicide attempts (aOR = 2.3; 95% CI: 1.63–3.25), prior suicidal thoughts (aOR = 2.88; 95% CI: 1.87–4.43), binge drinking (aOR = 1.52; 95% CI: 1.06–2.16), ever experiencing homelessness (aOR = 2.66; 95% CI: 1.69–4.18), experiencing a hate crime (aOR = 1.68; 95% CI: 1.2–2.36), engaging in sex work (aOR = 2.5; 95% CI: 1.69–3.72), ever experiencing verbal abuse or harassment (aOR = 3.57; 95% CI: 1.9–6.72), ever experiencing physical abuse or harassment (aOR = 3.72; 95% CI: 2.52–5.49), and injecting drugs in the past year (aOR = 1.79; 95% CI = 1.02–3.14). In multivariable models, condomless sex, number of sexual partners, ever being undocumented, and any substance use in the past month were not statistically significantly associated with sexual IPV (data not shown in the figure).

In addition, we examine evidence of interactions between the three most salient correlates (based on effect size), similar to a prior study (30). We observed that those who experienced physical abuse, verbal abuse, and had a history of suicide ideation had a 3.5-fold greater odds of sexual IPV (95% CI = 2.44–5.03), compared with those who did not report any of these correlates.

## Discussion

In this study, we observed a high prevalence of sexual IPV among trans women in San Francisco, with more than one in three (36%) trans women ever experiencing sexual IPV. In addition, we sought to examine correlates of sexual IPV. We observed that the correlates of sexual IPV in the present study are broadly consistent with the correlates of any IPV previously documented in a systematic review among trans people (8). These findings suggest a high concordance in the correlates of both IPV and sexual IPV among trans women. The high prevalence of sexual IPV also points to the need to develop effective screening tools for provider screening and referral for supportive services to address this specific type of trauma. Given the correlations between sexual IPV victimization and diagnoses for mental health conditions, mental health providers may be uniquely positioned to screen for sexual IPV, and studies have noted the value of providers' IPV screening for engaging people in interventions and improving safety (31). Nevertheless, more research is needed to develop interventions for mental health providers that are effective in enhancing their ability to screen and respond to sexual IPV (31).

We also found that lifetime experiences of sexual IPV were significantly higher among trans women who experience adverse mental health conditions (e.g., depression, PTSD, anxiety, suicidality) and markers of socioeconomic disadvantage and marginalization, including experiencing homeless, transactional sex, and a prior history of exposure to violence, harassment, and discrimination related to their gender identity and/or expression. These findings corroborate prior quantitative (8) and qualitative (11) literature reviews that have noted the unique vulnerabilities faced by trans women, including transphobia, stigma, and structural discrimination, which are also posited to contribute to trans women's social and economic isolation—factors that may either make them more vulnerable to sexual IPV or may co-occur with sexual IPV. These findings may be bidirectional. For example, these results may point to the negative mental health impacts of sexual IPV victimization in an extremely adverse risk environment due to the extreme marginalization trans women face because of their gender identity and expression (i.e., sexual IPV may be an exposure that can lead to negative outcomes). Alternatively, these correlates (poor mental health, homelessness, and past experiences of violence or discrimination) may also increase the risk of experiencing sexual IPV since these challenges can make it harder for trans women to access support and navigate relationships, as well as avoid unsafe situations. Above all, these data may point to an urgent need to create safety. One approach to safety for trans women is through economic opportunity so that trans women can have safe living situations and freedom from violence that often takes place in partnerships, be they interpersonal or commercial, with uneven power distributions (32). Policies are also urgently needed that protect trans women from violence, harassment, and discrimination in and outside of partnerships, ideally with approaches that focus on social inclusion in society at large (33).

In addition, we found an association between sexual IPV and health conditions and behaviors consistent with the SAVA syndemic framework. These findings underscore how sexual IPV is closely linked to other syndemic health conditions and point to the need for interventions and prevention that address multiple factors together. For example, interventions for trans women who survived sexual IPV may benefit from integrated approaches that attend to mental health, substance use, and HIV prevention needs of victims. Given these co-occurring epidemics' intertwined and synergistic effects, jointly addressing them in a multilevel, integrated fashion may amplify positive results (34).

Moreover, we found an association between sexual IPV and less optimal access to gender-affirming care, including inconsistent hormone use and the use of non-prescribed hormones. In another study conducted by our research group, we found that consistent hormone use was associated with a history of sexual violence among trans women (35). Taken together, these findings may point to either the additional negative consequences related to sexual violence and sexual IPV unique among trans women, specific to their ability to access gender-affirming services (i.e., exposure to sexual IPV can have negative impacts on gender-affirming care). Alternatively, it is also plausible that disruptions in access to gender-affirming care may be markers of broader structural barriers, which may, in turn, also lead to sexual IPV. Other research has studied the phenomenon of “passing” as key to safety for trans women (15). In this way, the correlation between disruptions in gender-affirming care and sexual IPV may also be bidirectional. Hence, in addition to remedying gender dysphoria, gender-affirming care may be a key intervention for violence prevention for trans women, while addressing sexual IPV among trans women may facilitate their ability to access gender affirming care.

## Limitations

Our study findings are subject to important limitations that should be considered. The self-reported measures in our study may be subject to social desirability bias and recall bias. Given the stigmatized and sensitive nature of sexual IPV, it' is plausible that our study reflects an underestimation of the true prevalence of this outcome. However, the use of standardized questionnaires and CAPI may have helped mitigate these potential threats to validity (36). Moreover, the relative consistency in the high prevalence of sexual IPV in our sample relative to those observed in the literature also provides us with confidence in our findings (8). Another limitation of our study is the cross-sectional nature and the lifetime assessment of sexual IPV. Hence, we are not able to ascertain the temporal sequence between our correlates and outcome, nor are we able to determine the directions of the relationships between our outcome of interest and correlates. Studies examining recent sexual IPV and time-varying correlates can further help fill the gaps in our understanding of factors linked to sexual IPV. Moreover, our study did not assess certain factors previously linked to IPV, such as disability status (9) or avoidant coping (22). Hence, we were not able to confirm whether these factors were also linked to sexual IPV in the present study. In addition, our models may not have accounted for all potential confounders, which may bias our findings. Larger studies with more robust measures of potential confounders may help elucidate the true underlying relationships between our correlates of interest and sexual IPV. Furthermore, the parent study used for this secondary data analysis had a primary aim of understanding HIV incidence among trans women and was not designed to study trans-specific IPV and sexual IPV in depth. We recognize that IPV and sexual IPV among trans women encompass a wider range of trans-specific issues and concepts, which were not measured in our study. For example, emerging data synthesized across qualitative studies point to novel themes on IPV among transgender individuals not reflected in the traditional understanding of IPV (11). Moreover, the study did not measure more nuanced dimensions of sexual IPV, including severity, perpetrator characteristics, and duration of abuse, which limits the depths of our analyses. Future studies among transgender individuals that examine these wider trans-specific IPV experiences, as well as specific sexual IPV dimensions, are needed to expand our understanding of IPV and sexual IPV in the literature.

## Conclusions

Our findings elucidate the extraordinary prevalence of sexual IPV experienced by trans women and demonstrate that there is a constellation of correlates of sexual IPV that trans women likely face before and after sexual IPV. Moreover, our study provides empirical data on the overlaps between factors and health consequences associated with IPV and sexual IPV. Numerous interventions are needed to prevent and address sexual IPV for trans women. IPV screeners in gender-affirming primary care and behavioral health, where many trans women access services, should be routine, given the high prevalence. Providers of gender-affirming care and mental health services for transwomen—including those addressing depression, PTSD, anxiety, and substance use disorder—could be trained to use validated screening tools for sexual IPV (37) and trauma-informed care to better support trans women who experienced sexual IPV (38). Ongoing care postscreening will need to address the range of negative health conditions that may likely co-occur among trans women who experienced sexual IPV, and addressing these multiple syndemic conditions with integrated approaches may be the most effective.

## Data Availability

The data analyzed in this study are subject to the following licenses/restrictions: Data are available upon request and with appropriate IRB approval. Requests to access these datasets should be directed to glenn-milo.santos@ucsf.edu.
